# Reasons for maternal near-miss in Bahir Dar city administration, northwest Ethiopia: a qualitative interview approach using socio-ecological model

**DOI:** 10.3389/fgwh.2025.1535379

**Published:** 2025-04-23

**Authors:** Yinager Workineh, Getu Degu Alene, Gedefaw Abeje Fekadu

**Affiliations:** ^1^ Department of Midwifery, College of Medicine and Health Sciences, Bahir Dar University, Bahir Dar, Ethiopia; ^2^ Department of Epidemiology and Biostatistics, College of Medicine and Health Sciences, Bahir Dar University, Bahir Dar, Ethiopia; ^3^ Department of Reproductive Health and Population Studies, College of Medicine and Health Sciences, Bahir Dar University, Bahir Dar, Ethiopia

**Keywords:** maternal near-miss, contributors, opportunities, socio-ecological model, Bahir Dar

## Abstract

**Introduction:**

Maternal near-miss means the experience of a woman who encounters complications of pregnancy, childbirth, or within 42 days of termination but survives. Maternal near-miss is common in developing nations like Ethiopia. Exploring healthcare system risk factors and opportunities informs policy, but understanding the complex contributors to maternal near-miss remains limited in the Ethiopian context. Therefore, this study aims to explore reasons using a socio-ecological model in Bahir Dar City, Ethiopia, 2023.

**Methods:**

A phenomenological study was conducted in the rural part of Bahir Dar city administration, northwest Ethiopia, from September 28th–December 10th, 2023. In-depth interviewees were women who experienced maternal near-miss. Key informants included husbands, women development army leaders, health extension workers, obstetric care providers, and health office holders. Participants were purposively selected until information saturation was reached, after interviewing twenty-five individuals. Data were collected using open-ended interview guides, with audio recordings and field notes. Verbatim transcription was conducted after each interview, and contextual translation was used to translate Amharic transcripts into English. Findings were made trustworthy through data triangulation, member checks, clear descriptions, and contextual translation. Data were analyzed using the framework analysis technique with Open Code 4.03, and results were reported within each theme.

**Results:**

Based on the Socio-Ecological Model, risk factors, protective factors, and strategies emerged from in-depth and key informant interviews. Individual-level reasons included poor knowledge, trust in traditional practices, and lack of decision-making power. Family-level contributors included male dominance, negligence, and disagreement. Organizational-level reasons encompassed non-compassionate care, resource scarcity, consultation delays, and lack of privacy. Community-level contributors included rumors, conflicts, transportation barriers, and harmful cultural practices. Public policy-level reasons were a lack of cascading protocols or guidelines and lengthy referral bureaucracy. Protective factors against maternal near-miss were identified at various levels, including self-care, acceptance of instruction, adaptation to modern healthcare, family trust-relationship, exempted services, mentorship, a three-tier healthcare system, and enhanced community engagement.

**Conclusion:**

Maternal near-miss was determined by complex contributors and opportunities at intrapersonal, interpersonal, organizational, community, and policy-level. We recommend addressing risk factors and utilizing potential opportunities to prevent maternal near-miss.

## Introduction

A maternal near-miss, also known as severe acute maternal morbidity, is characterized by a woman facing a critical complication that poses a life-threatening risk during pregnancy, childbirth, or within 42 days after delivery, but ultimately surviving (1).

The World Health Organization (WHO) introduced maternal near-miss statistics to identify life- threatening situations during pregnancy and childbirth (1). Maternal near-miss statistics provide a quicker way to identify health system weaknesses and prioritize maternal health issues than relying solely on maternal death data (1, 2). The WHO developed diagnostic tools for maternal near-miss to facilitate global implementation, including clinical, laboratory, and management criteria (3). These tools have also been adapted and validated for use in Sub-Saharan Africa (SSA) (4).

The 2024 WHO reports show that about 287,000 women died during or after pregnancy and childbirth in 2020 worldwide, with nearly 95% of these deaths in low- and lower-middle-income countries. Sub-Saharan Africa and Southern Asia accounted for 87% (253,000) of global maternal deaths, with SSA alone responsible for 70% (202,000) and Southern Asia for 16% (47,000) (5). In Ethiopia, the maternal mortality ratio (MMR) was 412 per 100,000 live births according to the 2019 Ethiopian Demography Health Survey (6), exceeding the global average MMR of 211 per 100,000 live births but lower than the MMR in SSA, which was 553 per 100,000 live births (7).

Maternal near-miss prevalence varies (0.80%–8.23% disease-specific measures, 0.01%–2.99% management-based criteria) (8). Globally, the ratio is 18.57 per 1,000 live births (9). Europe has the lowest ratio at 3.10 per 1,000 live births (9) while African and Asian middle- and low-income countries face the highest burden (10). In SSA, the ratio is 24.2 per 1,000 live births (11). In Ethiopia, the prevalence rate is 12.57%, with the highest rate in the Amhara region (26.5%) (12).

Maternal near-miss and deaths result from obstetric causes such as pre-eclampsia, eclampsia, obstetric hemorrhage, and puerperal sepsis (13). Globally, hemorrhage is the main cause of maternal mortality (14). In SSA, obstetric hemorrhage (28.8%) is the leading cause, followed by hypertensive disorders (22.1%), non-obstetric problems (18.8%), and pregnancy-related infections (11.5%) (15). In Ethiopia, severe postpartum hemorrhage (34.4%) and eclampsia (34.4%) are major causes (16). Maternal death and near-miss are influenced by complex risk factors, including delays at different levels (17), and a combination of sociocultural, political, environmental, organizational, psychological, and biological factors (18).

The Sustainable Development Goal aims to reach a maternal mortality ratio (MMR) of 70 per 100,000 live births by 2030 (19). Ethiopia also aims to reduce MMR to 255 per 100,000 live births by 2025 (20). However, progress towards this target in the Ethiopian context is lagging.

There are three levels of healthcare systems in Ethiopia: the first level providing primary healthcare through health posts serving 3,000–5,000 people and health centers for 25,000–40,000 people; the second level at district hospitals serving 100,000–150,000 people; and hospitals higher up in the city providing tertiary care to over one million people (21). Care cascading remains difficult despite an established referral chain, including from health posts to health centers and from there up to primary and specialized hospitals (22). In Ethiopia, caring systems are heavily influenced by cultural and gender dynamics that impact health-seeking behavior and limit women's care access (22). At a local level, communities, leaders, and networks are key for health service uptake (23). Although it is important to understand the reasons behind maternal near-miss in such complex interplay in Ethiopia, the majority of studies have concentrated on individual-level factors through quantitative approaches (12, 24–27). Hence it is essential to investigate the contributing factors across multiple levels using a specific model in the Ethiopian context.

The social-ecological model (SEM) is a comprehensive framework that recognizes the complex interplay between individuals and their environment (28). A qualitative approach using the SEM is crucial for exploring maternal near-miss reasons, as it captures the complex influences of social support, cultural attitudes, and healthcare access (29). Engaging women with their husbands and healthcare provider networks reveals the underlying causes and systemic difficulties that contribute to maternal near-miss. This study fills information gaps and provides an additional perspective by stressing social determinants using a holistic framework (30). Exploring different perspectives across multiple levels is necessary for improving decision-making and protecting the safety and well-being of pregnant women. This evidence will help to find workable solutions for maternal health challenges such as maternal near-miss. Hence, understanding the multidimensional contributors is vital for improving maternal health outcomes. Therefore, this study aimed to explore reasons for maternal near-miss using SEM in Bahir Dar City administration, northwest Ethiopia, 2023.

## Methods

### Study setting, and period

The study was conducted in Bahir Dar city administration, northwest Ethiopia from 28th September–10th December, 2023. Bahir Dar, the capital city of Amhara regional state, is found 484 km from Addis Ababa, the capital city of Ethiopia. It serves as the political, economic, and cultural hub of Amhara National Regional State. The city is situated on the southern coasts of Lake Tana at 11°36′ N latitude and 37°23′ E longitude. Bahir Dar city is divided into 6 sub-cities and four satellite towns. The city administration consists of 17 *kebeles*. *Kebele* is the smallest administrative unit in Ethiopia. Of these *kebeles*, four of them were rural. According to the City Health Department reports, the city has three governmental hospitals, eleven health centers, fifteen health posts, four private hospitals, fifty six private specialty clinics and thirteen private medium clinics (31).

### Study design

A phenomenological study was conducted to explore reasons for maternal near-miss across different actors. The philosophy of the phenomenological approach focuses on understanding consciousness and meanings drawn from human experiences (32). It discloses the essence of experiences while emphasizing individual outlook and interpretation (33). This research paradigm centers on intentionality, underlining that every experience targets something. It prioritizes description, reflecting human perception's depth and recognizing the influence of social and cultural contexts on experiences (34). This methodology is particularly effective when researching hard experiences (35). Understanding lived experiences is central to phenomenology as a philosophy and method. The research topic poses a “what” query about the phenomenon under study, and phenomenological research is appropriate for examining “how,” “what,” and “why” questions (36). This study involved an in-depth examination of the maternal near-miss within its network.

### Study participants recruitment

In this study, participants for in-depth interviews (IDIs) and key informant interviews (KIIs) were recruited. The IDI participants were women who had experienced maternal near-miss within the last six weeks, while the KII participants included their husbands, women development army leaders, health extension workers, obstetric care providers, and health office holders. The IDI participants were recruited using a health facility-to-community linkage approach, while the KII participants were recruited from the community and their workplaces.

### Sample size determination

We aimed to include 35 participants, with 5 individuals from each group, ensuring equal representation and facilitating a thorough exploration of diverse perspectives. Information saturation was reached after conducting interviews with 25 participants, including 5 women who experienced maternal near-miss, 4 husbands, 3 health extension workers, 3 women development army leaders, 4 midwives, 3 physicians, and 3 health office holders.

### Sampling technique

The study participants were selected using a purposive sampling method. The women who experienced a maternal near-miss were identified at the health facilities by communicating with care providers and reviewing their medical records. Their contact information, including their address and phone numbers, was obtained from them or their relatives. Midwives and physicians who provided care for the selected women were recruited in health facilities. Health office holders were also selected in the same health facilities. Following this, their husbands, the women development army leaders, and health extension workers from women's respective *kebeles* were also included as participants.

### Data collection tool and process

A face-to-face interview guide was utilized to collect data from the participants. To ensure ease of comprehension, the interview guide was initially developed in English and later translated into Amharic. The interview guide consisted of open-ended questions (Supplementary File S1). Depending on the participant's responses, probing questions were asked to dig out deeper into the topic. Participants were encouraged to provide more information by using prompts such as “could you tell me more about that?” or “could you provide an example of this?” A digital voice recorder was used to capture the audio after obtaining permission from each participant. The Sony ICD-BX140 digital voice recorder was used to record interviews in MP3 format. It has 4GB internal memory, supports flash drives, and features intuitive controls. Compact and lightweight, it operates on external batteries and offers high-quality stereo recording.

The interviewer introduced himself and provided ample details about the purpose of the study at the beginning of each interview. The interview commenced once the participant was at ease and some background data were gathered. The interview began with a broad and general topic, gradually transitioning into more in-depth inquiries. As the data collection progressed, the questions became more narrowly focused. During the interview, the participants were engaged and maintained interest through the application of active listening, patience, and flexibility. The interviewer carefully listened and paraphrased the respondent's statements to ensure a complete understanding of their intended meaning. Patience was exercised by allowing the participants to speak freely while gently guiding the conversation to cover important topics or refocusing it if it veered off track. The interviewer let the respondents share their perspectives and thoughts. Flexibility was demonstrated by the interviewer's willingness to accommodate slight deviations from the main topic, which may have required rearranging, or reordering the questions or introducing new ones. The interviewer asked each individual a question and attentively listened until they finished expressing their thoughts. Researcher interviewed two participants per day.

### Trustworthiness

Data validity was ensured through rigorous, thoughtful, and honest questioning, as well as by establishing a connection with the participants. The accuracy of the answers was verified through summarization and reflection of the participants' narratives. By utilizing the participants' own words, interpretive validity was maintained. The researchers read the data repeatedly until fully grasped its meaning. Overall, credibility, dependability, conformability, and transferability of the data were ensured through the application of various mechanisms.

### Credibility

Instead of using highly suggestive questions, open-ended questions were prepared and utilized. Participants were instructed to share their own ideas, motivations, or experiences rather than those of others. To assess the coherence of the respondents' viewpoints, any conflicting explanations or contradictory concepts that emerged during the discussion were carefully examined. The speakers were encouraged to provide specific examples rather than generalizing. Inconsistencies were investigated by triangulating the information gathered from respondents through different data collection techniques, including IDI and KII.

Overall, credibility was ensured through the iteration process, data source triangulation, inclusion of rich quotes, and member checks. We included the cyclical and flexible nature of data collection and analysis. This involved continuously refining methods by adapting questions based on initial findings and conducting follow-up interviews. We also embraced triangulation through diverse participant interviews by intentionally selecting participants from varied backgrounds. Furthermore, member checking was conducted to improve credibility by sharing the translated documents with participants to ensure their perspectives were accurately represented. The researcher translated the audio recordings into Amharic and read them to a non- literate woman and her husband. The translated documents were also provided to two health providers for feedback, enabling participants to clarify or add context.

### Dependability

The researcher's background, professional training, project-specific decisions in the field, and revisions made to the questions were transparently presented to showcase dependability. The research questions were formulated, with consistency across data sources and logical connections to the study's objectives and design. Modifying study procedures based on evolving findings was performed in this study. This adaptive approach allows researchers to refine methods, adjust interview questions, and involve participants for feedback, enhancing understanding and improving the credibility of the research. During the analysis phase, efforts were made to identify response patterns that were logically coherent and exhibited a reasonable level of stability over time.

### Conformability

By maintaining an audit trail and practicing reflexivity, the researcher ensured a clear distinction between their values and those of the study participants. This study used an audit trail that included complete records of study design, data collection, changes made, and critical decisions to rigorously document the process and increase credibility. The researcher allowed participants to freely express their experiences, values, and expectations without any constraints.

### Transferability

The conclusions were drawn with careful consideration, ensuring that they were supported by the available data. To maintain the transferability of the findings, the research setting, characteristics of the study participants, the nature of their interactions with the researcher, and the physical surroundings were clearly described.

### Data processing and management

Open-ended field guides, field notepads, and an audio recorder were utilized to capture the data. Interviews were prepared and organized for analysis for in-depth examination. Then the audios were transcribed into text format for accessibility. Reviewing and correcting transcripts for errors was performed. Researchers anonymized data by removing identifiable information to protect participants' privacy. Data was organized into segments by women who experienced maternal near-miss, their husbands, health extension workers, women development army leaders, midwives, physicians, and health office holders. Before formal coding, a preliminary coding framework was developed. The final data were stored on a password-protected computer in word-processing files. The translated data were then uploaded to the Open Code 4.03 program for management.

### Data analysis

The researcher immediately began writing notes after each interview. These notes included relevant information about the interview itself, environment, participant's health or behavior, and researcher's impressions. Interviewer transcribed the interviews in their original language (Amharic) after each day's interview. The audio was transcribed using a verbatim approach, capturing the exact words spoken. The transcription included anonymized names of the interviewer and interviewee, date and time of the interview, location, speaker attribution (indicating who said what), and time stamps. In cases where the audio quality was poor or the discussion required clarification, the transcription process involved adding explanatory comments, marking unclear or missing audio with ellipses, and highlighting specific words or phrases.

The researcher conducted the translation of the transcript from Amharic to English. The researcher verified the meanings of certain local languages to avoid any misinterpretations during the translation process. Conceptual translation was employed, which involved multiple readings and reviews of the transcribed audio to ensure the accuracy of the wording and the authentic representation of the participant's experience.

The data was analyzed using the framework analysis method based on the SEM, which explains how health behaviors are shaped by the complex interactions between individuals and their environment, including factors at the individual, interpersonal, organizational, community, and policy levels (37). The authors followed a six-step approach: (1) familiarize with data, (2) generate initial codes, (3) search for themes, (4) review themes, (5) define themes, and (6) write-up.

The first step is to become familiar with the data by repeatedly reviewing the recordings and transcripts. The second step is to generate initial codes, organizing the data meaningfully and reducing it into smaller segments using an open coding approach. The third step is to search for themes, identifying connections between concepts and grouping codes into overarching themes. The fourth step is to review and refine these preliminary themes. The fifth step is to define the themes, identifying the core essence of each one. The final phase is to write the report, providing a concise, coherent account of the data's story across the themes, supported by evidence and examples.

## Results

### Background characteristics

The study encompassed individuals aged 28–48 years. The study participants included five women who experienced maternal near-miss, four their husbands, three health extension workers, three women development army leaders, four midwives, three physicians, and three health office holders (Table 1).

**Table 1:** Participants' characteristics in Bahir Dar city administration, northwest Ethiopia, 2023.

Characteristics	Response	Number
Participant	Women	5
	Husband	4
	Health development army	3
	Health extension workers	3
	Midwifery	4
	Physician	3
	Health office holders	3
Age	25–35 year	7
	36–45 year	14
	46 year and above	4
Residence	Urban	11
	Rural	14
Sex	Male	10
	Female	15
Educational status	Non-educated	9
	Primary	3
	Secondary	1
	College and above	12

### Reasons of maternal near-miss

The analysis of maternal near-miss incidents was framed using the SEM, examining factors at the individual, interpersonal, organizational, community, and policy levels. Within each of these levels, the data was further categorized into three sub-themes: risk factors, protective factors, and strategies.

### Theme 1: individual-level

At the individual level, risk factors, protective factors, and strategies associated with maternal near-miss incidents were identified.

#### Sub-theme 1: risk factors

Some participants reported that individual-level factors such as poor knowledge, low income, traditional practices, lack of decision-making, fear, workload, misconceptions, past experiences, and religious beliefs influenced maternal near-miss incidents.

Lack of knowledge about pregnancy complications contributed to reduced healthcare-seeking and maternal near-miss. For example, one respondent stated: *“They (mothers) considered the normal previous pregnancy outcomes as a guarantee for the current and next one. And the first and huge gap is the perception of having previous normal outcome as a guaranty for the current pregnancy. Therefore they (mothers) said that why I am going to there?”* (Male, 40 year old, midwife)

Low household income also contributed to maternal near-miss. A participant described: *“I do not have enough money. When the bleeding started, I did not go to the hospital because I do not have money for contract transportation at night time.”* (Female, 37 year old, woman experienced near-miss)

Trust in traditional practices facilitated maternal near-miss. For example, one respondent explained: *“When the delivered mother faced bleeding, they practiced different traditional practices like immersing the woman in water, move the mother in different direction like up and down by putting material underneath assuming the placenta will be move downwards.”* (Male, 40 year old, midwife)

Lack of decision-making power affected women's autonomy, leading to seeking approval from others, which facilitated maternal near-miss. One participant stated: *“He (husband) decides when the problem happens. If he is not here, I wait him until he returns. Then I tell when he returns and he decides.”* (Female, 39 year old, woman experienced near-miss)

Workload also facilitated maternal near-miss. *“In rural area, they (mothers) will come to home at the same time with their husbands from their farm by equally doing the tasks. She comes to with husband and she works at home. She performs all the housework. She takes cares of children.”* (Male, 28 year old, midwife)

Fear was a risk factor for maternal near-miss, undermining women's confidence. For instance, a participant said: *“Oh, she doesn't even talk to health professionals. Even if s/he (health professional) talks to her now, she turns away and cries, she doesn't talk with them.”* (Male, 44 year old, husband)

Misconceptions about pregnancy and related issues were also a risk factor for maternal near-miss. In this regard, a participant described: *“Well, there are people who use term like* ‘*serakian*’ *for puerperium woman. When a mother bleeds after giving birth, accept it as normal and think that it is right.”* (Male, 28 year old, midwife)

Religion belief can impact maternal health outcomes. A person stated: *“Indirectly, for example, you can consider take birth control. There are religions that do not promote birth control. The more she gives birth to more than 6 or 7, the more likely she is to have this kind of problem.”* (Male, 28 year old, midwife)

Past bad experiences were a risk factor for maternal near-miss. For example, one participant described: *“Two neighbors might be pregnant. Unfortunately, it may be by chance, or lateness, or severe condition, that one mother may not be saved even if she gains a service in health facility. If she passes away with that kind of experience, the community argues why not I just sit at home and die, if someone else dies in the facility after gaining the service?”* (Male, 40 year old, midwife)

#### Sub-theme 2: protective factors

Protective factors against maternal near-miss at the individual level included previous positive experiences, self-care, self-confidence, social support, acceptance of medical advice, adoption of modern healthcare, and improved desire to seek maternal services.

Prior positive experiences were particularly protective, providing fresh ideas and strong coping skills. For example, one participant described: *“Exposed mothers have a high commitment to the future. For example, their frequency to visit the health facility increases.”* (Male, 42 year old, midwife)

Self-care was a protective factor against maternal near-miss, promoting good mental health and reducing pregnancy complications. For instance, one participant stated: *“Not doing hard work; hourly feeding; taking a shower; taking and applying what she has received from the government are good.”* (Female, 47 year old, women development army leader)

Self-confidence was a protective factor, enhancing women's abilities through self-acceptance, trust, and self-control. A participant explained: *“As an individual, I have inherent value and dignity. I am sick, I respect my self-respect and I try to avoid humiliation. In times of crisis, I would without a doubt seek medical attention from a physician.”* (Female, 41 year old, woman experienced near-miss)

Social support also protects against maternal near-miss, helping women embrace their pregnancies within their communities. In this regard, one participant stated: *“As we see in our area after she is pregnant, she will be helped. In one way or another, they prevent women from doing hard work. They give her rest. They assume that she will give a child who is going to give grace.”* (Male, 42 year old, midwife)

Acceptance of medical instructions, through a professional-patient partnership, protected against maternal near-miss. For example, a respondent explained: *“I accepted the things that they informed me. For example, I did not do hard work; I keep my hygiene; I eat the recommended foods. I went to the health center on the indicated time.”* (Female, 38-year-old, woman experienced near-miss)

Adaptation of modern healthcare was protective, increasing health-seeking behavior and service use, reducing pregnancy complications. *“You don't say that everything is similar with the previous. It is the time of civilized. At this time, by thinking there are no people who do not go to health institution, everyone is trained. I don't think anyone would do that nowadays except maybe old mothers.”* (Male, 43 year old, husband)

#### Sub-theme 3: strategies

Strategies to address individual factors include risk reminders and community health education to reduce maternal near-miss. Risk reminders were used to implement interventions targeting health-risk behaviors of pregnant women. For instance, one participant stated: *“…risk reminder will be given. I have seen from my experience that number of institutional deliveries will be higher due to given risk reminder.”* (Male, 42 year old, midwife)

Community health education addressed different factors to prevent and mitigate maternal near- miss. *“I provide health education. I give this house-to-house visit. If I get pregnant woman, I advise her to visit health facility.”* (Female, 33 year old, health extension worker)

### Theme 2: interpersonal-level

At the interpersonal level, there were both risk factors and protective factors that influenced maternal near-miss.

#### Sub-theme 1: risk factors

Interpersonal risk factors like male dominance, lack of spousal support, and disagreements contributed to delays in maternal care. A participant explained: *“Indeed, sometimes there is a saying called a woman knows, a man finishes. It is the man who makes the decision in the end after the women suggested.”* (Male, 43 year old, husband)

Negligence also contributed to maternal near-miss by failing to provide proper care. For example, a participant stated: *“Negligence is sometimes one of the processes we observe. Mothers who have no previous experience and have given normal birth once or twice in their home, think that there is no problem in this pregnancy.”* (Male, 42 year old, midwife).

Family disagreements were an interpersonal risk factor for maternal near-miss. For instance, one respondent explained: *“…there are husbands and wives who are quarreling. Now what are they doing? What is with them? If they discuss about the issue, consensuses will come. But why do they fight? If not, peaceful separation is there.”* (Male, 44 year old, husband)

#### Sub-theme 2: protective factors

Protective interpersonal factors included family trust, spousal support, shared decision-making, and positive parental involvement. Strong family trust and communication helped safeguard against maternal near-miss. For example, one person stated: *“I think that the more the social bonding is, the more the woman's family is concerned about the life of the woman, and because they think that the problem is ours too.”* (Male, 28 year old, midwife)

Spousal support provided benefits like improved maternal health, reduced complications, and enhanced nutrition. In this regard, a respondent stated: *“I support her as much as she cares for me and I support her as much as I can. It is necessary that I fulfill what she wants in terms of work and other needs.”* (Male, 43 year old, husband)

Shared decision-making between spouses was a protective factor, enabling collaborative problem-solving on maternal health issues. For instance, one respondent stated: *“Of course, since this child is our first child. Even if we don't have children previously, it is decided by discussing together the events.”* (Male, 41 year old, husband)

Acceptance of medical referrals within the household was another protective factor. For example, a participant explained: *“Now, in order to prevent this situation from reaching the point of death, it is one thing that they have a commitment of accepting of referral at the house hold level.”* (Male, 40 year old, midwife)

Positive parental involvement prevented maternal near-miss. In this regard, a person stated: *“I consider it as good when she is with their parents when delivery time is arriving.”* (Male, 45 year old, physician)

#### Sub-theme 3: strategies

Caring strategies like expressing wishes, preparing food, visiting, and sharing work prevented maternal near-miss by strengthening family bonds and the woman's psychology. For example, one respondent stated: *“Things like Mariam's asks, Mariam's bird, and making porridge and calling it as a baby shower after 7 months of pregnancy is something that is encouraged in urban areas, because remembering pregnancy, expressing good wishes, means that there will be an opportunity to help her to have a better health condition.”* (Male, 42 year old, midwife)

### Theme 3: organizational-level

The study indicated that organizational risk and protective factors as well as strategies of maternal near miss in health facilities.

#### Sub-theme 1: risk factors

Organizational risk factors included non-compassionate care, resource scarcity, negative experiences, lack of privacy, night service provision, and professional-client disproportion. Non- compassionate care lacked empathy and efforts to alleviate women's suffering. For example, a respondent described: *“And I do not want to mention the name of hospital. In this hospital when the mother was in second stage, he forced her to push. She says I can't. And when he gives funds pressure, she returned her face towards to him. Then he punched her.”* (Male, 40 year old, midwife).

Resource scarcity reduced access to maternal healthcare, increasing risks of complications like bleeding and infections during childbirth. For instance, one participant explained: *“The professional does not have the knowledge and the commitment. For example, if you give birth in a hospital, the required professional and materials and supplies are not available there.”* (Male, 47 year old, husband)

The scarcity of resources affects the exempted services. One participant described: *“Nowadays mothers buy most things outside. I think it shows the weakness of the health institution. If it is free, the necessary materials should be obtained first within the health facility.”* (Male, 30 year old, midwife)

Negative healthcare experiences contributed to delays and maternal near-miss. One respondent stated: *“Primary, one-person facial expression is better than what s/he actually provided services. To be seen by someone else, they said that we prefer to death. And I notice that there are mothers who come to get the service saying that it is not because of the good service, but because of the good approach of the individual.”* (Male, 42 year old, midwife).

Role overlap was also another risk factor for occurrence of maternal near-miss in health organization. For example, a respondent described: *“And there is a tendency to role overlap, like midwifery and resident. This is the institutional problems which is associated with not properly distinguishing the role.”* (Male, 40 year old, midwife)

Delays in the consultation hierarchy reduced appropriate care and increased maternal near-miss. For instance, a participant explained: *“When we look at treatments, it goes to the intern and then the resident and then the senior. Based on time, when there is emergency situation, the most senior will be consulted. Sometimes there is delay; it is difficult to say that a senior is stand-by.”* (Male, 40 year old, midwife)

Lack of privacy was another risk factor at the institutional level. In this regard, a participant described: *“They (mothers) frustrated in association with the lack of privacy that we provide. It is believed that one mother should have her own room to lobar and deliver privately. Here now there are many people try to labor and deliver in one room.”* (Male, 42 year old, midwife)

Service provision at night time was also identified risk factors for maternal near-miss. *“Senior professionals cannot come at night for consultation. And that is the biggest problem. Second, there will be fatigue on night time. This care cannot be given formally. Sometimes it is difficult for you to manage multiple cases there due to limited manpower.”* (Male, 42 year old, midwife)

Additionally, lack of guideline adherence was a risk factor for maternal near-miss. For example, one participant explained: *“I think we are doing good to reduce maternal mortality. But there is a problem to perform the job stick with a guideline.”* (Male, 40 year old, midwife)

Last but not least institutional level risk factor was professional-client dis-proportionality. *“Well, I think that the number of mothers going to health facilities and professionals in our country is not proportional. A professional manages a huge number of mothers who come to the health facility, and this leads to fatigue.”* (Male, 30 year old, midwife)

#### Sub-theme 2: protective factors

Organizational factors like responsive professionals, exempted services, mentorship and training protected against maternal near-miss. For instance, health professionals' responsiveness shapes positive patient perceptions and utilization. In this regard, a participant described: *“She did not face the problem there, but I can't pass without thanking doctors. The doctors, up to now, they help her as long as they can secondary to God. And they spend the night taking care of what is just there from the beginning to the end.”* (Male, 41 year old, husband)

Provision of exempted maternal health services was a protective organizational factor, as fee exemption increased utilization of necessary care. For example, a respondent explained: *“It's free. Most of the women would have died if the services were not free, because most of the Ethiopian society is from poor rural area.”* (Female, 35 year old, woman development army leader)*.*

#### Sub-theme 3: strategies

Strategies like audits, case discussions, and teamwork were used to prevent maternal near-miss. Maternal near-miss and death audits reviewed cases to identify underlying factors and generate prevention recommendations. *“By conducting maternal death audit, maternal death audit indicates that the cause of one mother's death will be an intervention for 10 mothers' death, and a report will be made.”* (Male, 42 year old, midwife)

Morning case discussion is another protective factor of the occurrence of maternal near-miss. For example, a respondent stated: *“The discussion is made on maternal near-miss to prevent the same scenario from happening a second time. Case discussion is a major part of the learning process. I would say it is the main opportunity in this area.”* (Male, 40 year old, midwife)

Teamwork was a protective organizational factor, as collaborative efforts prevented maternal near-miss. For example, a participant described: *“There is also a process of working as a team with the related bodies of anesthesia for emergency situations. And after arriving this level as an institution, I think it's good to try to work with team coordination, for things that sometimes require attentions.”* (Male, 42 year old, midwife)

### Theme 4: community-level

At the community-level, there were cultural and physical environments that encompass a mother. Within this context, cultural values or norms can be considered as risk and protective factors as well as strategies of maternal near-miss within social networks.

#### Sub-theme 1: risk factors

Community-level risk factors included conflict, transportation barriers, lack of health support, beliefs, rumors, and harmful practices. For instance, a conflict in community was explored as risk factor. A participant stated: *“Not being able to access foods that pregnant mother's need, that they access other times. In addition, the ability to go to a health facility was stopped. This is what happens during conflict.”* (Male, 30 year old, midwife)

The transportation barrier in rural communities hinders physical access to maternal healthcare services, leading to severe complications. For example, one respondent said: *“For example, sometimes transportation is one of the problems. Transportation and access may not be available. Another thing is that if you have an ambulance phone, you may encounter something like this on the roads of transportation.”* (Male, 28 year old, midwife)

The lack of health extension workers support was also explored as a risk factor severe maternal morbidity. In this regard, a participant said: *“They (heath extension workers) did not come early in the morning. It started at 3 o'clock and then they leave at 5:30 by saying it is time for lunch. So, we cost for taxi for the second-round transport.”* (Male, 44 year old, husband)

Rumors in the community caused service utilization delays and complications. For example, a respondent stated: *“Doubtful truth itself is the challenge. Doubtful truth means a rumor that circulates in a society without verification. Rumors, they do cold-back. Individual may also refuse to be check by someone else for their health.”* (Male, 40 year old, midwife)

Harmful traditional practices were also the risk factors of maternal near-miss. In this regard, a participant described: *“There are traditional practices such as female genital mutilation and early marriage that leads to near to death.”* (Male, 35 year old, health extension worker)

#### Sub-theme 2: protective factors

Protective community factors included health programs, beneficial cultural practices, acceptance, engagement and cohesion. The availability of the health development army was a protective factor against maternal near-miss. In this regard, one participant stated: *“The so-called health development army help pregnant mothers. They facilitate the antenatal care use. They tell them what to do and what not to do. They tell them where they should give birth. They inform them. They tell them what to do. After giving birth, they audit them.”* (Male, 40 year old, midwife)

Beneficial cultural practices like preparing transport, making food, and giving gifts were protective community factors against maternal near-miss. For instance, a participant explained: *“Things like Mariam asks, Mariam's bird, and after 7 months of pregnancy, making porridge and calling it a baby shower, something that is encouraged in urban areas, because remembering pregnancy, expressing good wishes, means that there will be an opportunity to help her have a better health condition.”* (Male, 40 year old, midwife)

Community acceptance and cohesion was the protective factors of maternal near-miss. *“In our area, there is a strong social bonding. When she comes back to house, different things will be prepared. We received her by happiness. There is porridge, everything is there.”* (Male, 44 year old, husband)

#### Sub-theme 3: strategies

Social education campaigns were strategic mechanisms to prevent maternal near-miss at the community level. For example, a respondent stated: *“There may be provision of repeated awareness creation in order to accept the above packages and come to the health facility.”* (Male, 40 year old, midwife)

### Theme 5: policy-level

The study indicated that policy level risk and protective factors as well as strategies of maternal near-miss in the implementation the designed policies.

#### Sub-theme 1: risk factors

Policy-level risk factors included lack of local evidence, lengthy referrals, and insufficient guideline dissemination. The lack of incorporating local evidence in national protocol/guideline development was a policy-level risk factor. A participant stated: *“There are many maternal health issues explored by research. But those papers have not grown into policies.”* (Male, 28 year old, midwife)

Lengthy referral systems were a policy-level risk factor that delayed access to appropriate care. For example one participant explained: *“I don't think the referral mechanism is healthy. Because what do you think right now, direct referral from the health center to the primary hospital, general hospital is correct. Now, if it is specifically related to near-miss, it can be sent directly to the tertiary level from each level of health center.”* (Male, 40 year old, midwife)

Inadequate guideline/protocol cascading was a policy-level risk factor. In this regard, one respondent expressed: *“If there are protocols that can be changed, it is important to give training, explain the things that are changed, but it did not apply in our context.”* (Male, 30 year old, midwife)

#### Sub-theme 2: protective factors

Protective policy factors included social media, exempted services, three-tier healthcare system and international funds. The availability of social media like Telegram protected against maternal near-miss by disseminating guidelines. For example, a participant declared: *“The health office dispatch direct downward. And* via *Telegram Great Resource such updated protocol, guide lines were uploaded. We have it everywhere, that's where every guideline is in the store in every place.”* (Male, 40 year old, midwife)

Policy-level exempted service provision protected against maternal near-miss. Exempted medical and transportation services protected against maternal near-miss. For instance, one respondent stated: *“There is what we call exempted services is designed for maternal health. This one is a policy direction.”* (Female, 39 year old, health office holder)

Additionally, another participant elaborated: *“There is exempted program at policy level. For example, the assignment of an ambulance to a transport service is one of the strongest for the maternal health.”* (Male, 47 year old, physician)

Availability of international funds for maternal health was the protective factor of maternal near- miss. For example, one participant described: *“Another focus given for mothers is that the international funds themselves are very good.”* (Male, 28 year old, midwife)

#### Sub-theme 3: strategies

Strategies at the national level included connecting communities, strengthening rural institutions, and increasing hospital referrals through health workers and centers. For instance, a participant stated: *“In the countryside, there is a policy to connect one kebele with others.”* (Male, 42 year old, midwife)

Strengthening rural health and increasing hospital referrals through health workers were strategies to prevent maternal near-miss. In this regard, a participant explained: *“Our country already developed road map to increase the number of health facility in rural part. And they have tried to strengthen them. There is also a referral connection from health post to health center and then hospitals.”* (Male, 46 year old, physician)

**Figure 1:**
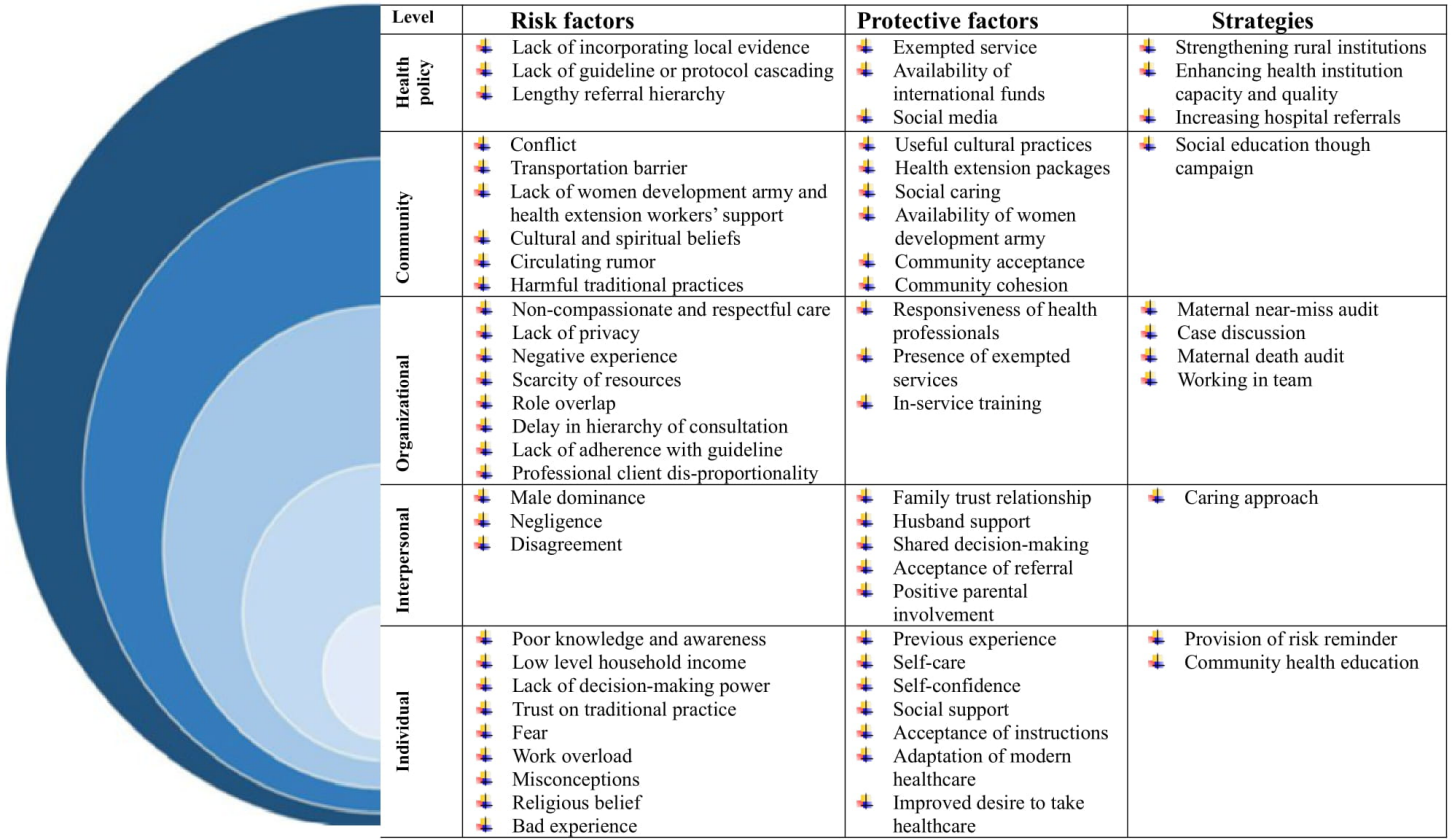
Risk and protective factors, and strategies of maternal near-miss in Bahir Dar city administration northwest Ethiopia, 2024.

The study identified various concepts at different levels. The researchers presented a model outlining the themes and categories within each theme for women who experienced a maternal near-miss event (Figure 1).

## Discussion

The reasons for maternal near-miss were explored through a qualitative approach in Bahir Dar, northwest Ethiopia using the SEM at multiple levels. The results from each level were illustrated through the lens of the three-delay model, which encompasses recognizing problems and deciding to seek care (delay one), transportation to reach appropriate care (delay two), and receiving proper care at the health facility (delay three) to indicate the interplay between social determinants of health and the specific barriers associated with each delay (17).

### Delay one

The first delay in healthcare access occurs when individuals recognize a problem but do not seek care owing to unawareness of symptom severity, cultural beliefs, or ambiguity about treatment options (38). In this regard, the current study explored contributors to the first delay that results in maternal near-miss at individuals (lack of knowledge and awareness, trust in traditional practices, misconception, limited decision-making power, and religious beliefs are individual- level contributors), family (male dominance, lack of support, and disagreements) and community (circulating rumors) levels.

Lack of knowledge and awareness contributed to delays leading to maternal near-miss in this study. Past evidence also indicated knowledge was a crucial individual-level factor influencing health behaviors (39). Understanding obstetric risks is vital for women and families during pregnancy, delivery, and postpartum (40). However, women's lack of health knowledge delays their recognition of when to seek medical care, hindering timely action and worsening health outcomes (41). Targeted education and awareness campaigns are essential to bridge these knowledge gaps and enhance healthcare access.

Misconceptions about health and treatments profoundly contribute to delay one in healthcare, leading to maternal near-miss in this study. Individuals often underestimate symptoms, believing they are not serious enough for care (42). Additionally, the belief in self-treatment leads to reliance on home remedies, while fear of serious diagnoses and cultural misunderstandings further delay timely medical intervention (42). Building trust in the healthcare system is essential, as misconceptions can erode this trust. Planned educational interventions and community outreach programs are necessary to clarify misconceptions and encourage women to seek timely care.

Trust in traditional practices affects how people recognize their need for medical care in the present study. These practices are associated with individuals with low socioeconomic status who often follow cultural practices, beliefs, and rituals without relying on scientific evidence (43). Many individuals tend to favor traditional healers instead of modern practitioners, which can impede the identification of serious health issues (44). Additionally, cultural beliefs influence health perceptions, causing some to regard certain symptoms as normal or not urgent (43). Targeted education is required to educate communities about the dangers of traditional practices and the value of medical treatment. Integrating culturally acceptable behaviors into medical procedures can foster trust and promote safe birthing. Community leaders should take part in these initiatives.

Lack of decision-making power expedited the occurrence of maternal near-miss. Women's limited decision-making power, influenced by family, diminished their use of healthcare services (45). Limited decision-making power negatively impacts maternal health by restricting access to reproductive services (46). Empowering women in healthcare decision-making can enhance outcomes and prevent maternal near-miss. Policymakers should promote gender equality, strengthen women's rights, and engage communities to support women's autonomy in health decisions.

Religious beliefs impact individuals' recognition of the need for medical attention, contributing to maternal near-miss incidents in this study. Many prioritize faith healing over conventional treatments, leading to delays in care (47). This further can lead to maternal near-miss by influencing healthcare decisions and emphasizing spiritual over medical treatment. To promote medical care while respecting spiritual beliefs and guaranteeing safe maternal health practices, culturally relevant education could be provided in collaboration with religious leaders.

The woman's family and cohabitants play a crucial role in shaping her maternal health decisions. According to social behavior theory, perceptions of others' actions and expected outcomes influence one's behavior (48). For instance, men hold ultimate authority over family planning, reproductive health, and allocation of healthcare resources (49). Male dominance in healthcare leads to delays in seeking care by restricting women's autonomy (50). Hence education programs empowering women and policy strategies addressing gender inequality are essential for improving health decisions and outcomes.

Support from family members is essential in a woman's health-seeking behavior, emotional well-being, and overall maternal health outcomes (51). Without adequate family support, women may experience maternal near-miss by delaying access to care, increasing stress and anxiety, impeding decision-making, and affecting mental health (52). Enhancing family and community support networks is essential for tackling these challenges and fostering better maternal health outcomes.

A family disagreement greatly affects how people recognize their need for medical care in the present study. Conflicts over healthcare decisions in the family can lead to delays and poor management (53). Disagreements among family members over obtaining medical care during pregnancy might cause delays in treatment. These disputes are caused by differing views, financial concerns, or cultural conventions, all of which affect healthcare-seeking behavior, support systems, and mental health (54). As a result, improving maternal health outcomes requires addressing such issues through education and family-centered care initiatives.

Circulating rumors were explored as the community-level contributors to the first delay that resulted in a maternal near-miss. Rumors about health risks may lead individuals to underestimate symptoms, and also cultural stigma around healthcare can prevent women from recognizing the urgency of their conditions (55). Additionally, rumors favoring faith healing over conventional treatments can result in critical delays in seeking care (56). High-profile figures may also spread skepticism about medical interventions, influencing their followers to postpone necessary healthcare based on misleading information (55). Addressing circulating rumors is crucial for improving maternal health outcomes, requiring public health campaigns to dispel myths and provide accurate information for informed decision-making.

### Delay two

Delay two involves challenges in accessing transportation to healthcare facilities after recognizing a health issue, significantly affecting health outcomes, especially in emergencies (17). Key factors include geographical barriers in remote areas, financial constraints, unreliable public transport, and lack of awareness about transportation options (38). The present study also indicated that community-level factors such as transportation barriers and conflict challenges contribute to the second delay that results in maternal near-miss. In developing countries such as Ethiopia poor roads and limited transport options make it difficult to reach specialized healthcare in rural areas (57), increasing the risks of maternal near-miss. To address such issues, strategies include improving infrastructure, establishing community transport programs, subsidizing costs, deploying mobile health units, and utilizing telemedicine.

In the present study, conflict was recognized as a community-level issue that contributed to the second delay, obstructing transportation access and leading to a maternal near-miss. This can happen due to destroyed infrastructure, insecurity deterring travel, financial constraints limiting affordability, and disrupted public transport systems (58). To tackle such problems during the conflict, solutions involve creating emergency transport services for pregnant women, securing safe passage for medical staff, and directing humanitarian aid toward maternal health needs.

### Delay three

Delay three refers to the challenges individuals face in receiving adequate care after reaching a healthcare facility, stemming from factors like care quality, healthcare personnel skills, and systemic issues (17). This study also revealed that the reasons for this third delay, which contributed to maternal near-miss, at organizational (non-compassionate care, resource scarcity, role overlap, delay in consultation, and non-compliance with clinical guidelines) and policy (lack of consideration of local evidence, lengthy referral system and lack of cascading protocol or guideline) levels.

Non-compassionate and disrespectful care by healthcare providers led to a third delay that resulted in a maternal near-miss. Disrespectful care during childbirth discourages women from seeking care and creates barriers to quality care (59). Disrespectful care can include physical abuse, lack of consent, confidentiality violations, undignified treatment, discrimination, neglect, lack of empathy, and unjustified confinement (60). Women who face disrespect during childbirth are less likely to seek facility-based care later, leading to higher maternal and perinatal mortality rates. Negative experiences create distrust in healthcare systems, adversely impacting health outcomes for mothers and infants (61). Addressing these challenges through training and adapting respectful care practices is critical to improving maternal health outcomes.

Insufficient organizational resources further contribute to the delay three and intern maternal near-miss. Lack of resources like space, staffing, and supplies creates barriers to providing appropriate care (62). The quality of care during childbirth depends on the facility's physical infrastructure, supplies, management, and staff competence (63). Inadequate resources lead to delays and compromised care. Timely access to emergency obstetric care and proper resources is crucial. Investing in healthcare infrastructure, resources, and staffing can reduce maternal near- miss.

Role overlap among healthcare providers significantly contributes to the third delay in emergency obstetric care in the present study. Ambiguity in responsibilities can lead to confusion and delays in care, while inefficient communication hinders timely treatment (64). Inadequate training and resource strain further exacerbate wait times, resulting in a chaotic environment that makes women feel neglected, ultimately fostering distrust in the healthcare system (65). Clearly defining responsibilities, enhancing training, and improving communication are crucial for reducing the third delay in emergency obstetric care.

The delay in consultation hierarchy between junior and senior staff within a healthcare facility significantly contributes to the third delay that resulted in maternal near-miss in this study. Hesitation to escalate urgent cases, unclear roles, inefficient triage processes, and poor communication can prolong decision-making and treatment initiation (66). This results in women feeling neglected, ultimately fostering distrust in the healthcare system and deterring them from seeking timely care. To address these issues, it's essential to improve communication, clarify roles and responsibilities, and empower junior staff to escalate urgent cases.

Non-compliance with clinical guidelines in emergency obstetric care significantly contributes to the third delay in the current study. This can be a result of inadequate training, poor resource availability, ineffective communication, and hierarchical barriers (67). These factors cause delays in decision-making and care provision, compromising quality and leading to maternal near-miss events (68). Improving training, ensuring resource availability, enhancing communication, and fostering a supportive environment can ensure compliance with clinical guidelines and improve maternal health outcomes.

Public policy is essential in addressing the third delay in emergency obstetric care by strengthening healthcare infrastructure, enhancing training, improving communication, ensuring resource allocation, and implementing monitoring systems (67). However, the current study has uncovered various challenges. This study explored the lack of consideration of local evidence in the maternal healthcare system can contribute to the third delay. This oversight leads to inadequate training, poor communication, and insufficient resource allocation, ultimately affecting maternal health outcomes (69). Without relevant local data, healthcare providers are ill- prepared to manage emergencies effectively, perpetuating delays in care provision and compromising patient safety (70). Hence incorporating local evidence into healthcare practices and policies is crucial for improving maternal health outcomes.

The lengthy referral system contributes to the third delay in receiving appropriate medical care in this study. Complex referral processes require patients to navigate multiple facilities, often leading to confusion and delays (71). Additionally, geographic barriers in rural areas increase travel times, exacerbating delays and risking complications during transit. Improving maternal healthcare pathways is essential to mitigate the negative effects of lengthy referral systems. This includes enhancing communication, streamlining referrals, and ensuring timely access to care.

The absence of a dispatching protocol was explored as the reason for delay in receiving appropriate medical care in this study. The absence of a dispatching protocol leads to inefficient patient management, longer wait times, and poor communication among healthcare staff regarding patient needs (72). These problems lead to inconsistent care, treatment delays, and increased risk of maternal near-miss (73). Establishing easily dispatching system protocols and guidelines is essential for improving maternal healthcare. Standardized procedures can enhance the efficiency of care delivery, ensure timely interventions, and ultimately reduce the occurrence of maternal near-miss.

The study had certain limitations. Even if we encouraged participants to express their thoughts freely by ensuring confidentiality and anonymity, using open-ended questions and multiple data sources, social desirability bias can occur due to the desire of participants to provide socially acceptable responses. Recall bias can also occur when participants struggle to remember past events accurately even if the women were interviewed immediately after discharge at the time they felt comfortable at their homes.

## Conclusions

Maternal near-miss cases are influenced by risk factors at individual, interpersonal, organizational, community, and policy levels. Cultural and social challenges within communities influence maternal near-miss incidents. Addressing maternal near-miss requires multilevel protective strategies. Hence, this emphasizes the significance of adapting a comprehensive, multi-level, and multi-stakeholder approach when planning, designing, and implementing interventions.

## Data Availability

The original contributions presented in the study are included in the article/Supplementary Material, further inquiries can be directed to the corresponding author.
