# Supplementary material for: Reasons for maternal near-miss in Bahir Dar city administration, northwest Ethiopia: a qualitative interview approach using socio-ecological model
Source: Front Glob Womens Health. 2025 Apr 23;6:1535379. doi: 10.3389/fgwh.2025.1535379 (PMC12055808; doi:10.3389/fgwh.2025.1535379)
Supplement: Supplementary file 1 [file Table1.docx]

**Additional file 1: Interview Guide**

1. **women, husband, and women development army**
2. How do you express the problem you/her faced?
3. Please tell me the factors that you believe to bring this problem?
4. What are the individual level factors do you think are related with near to death experiences in this pregnancy?
5. Describe the individual level strength/opportunity that support you to overcome the problem?
6. Please explain the interpersonal factors that you believe for occurrence of near to death?
7. Please tell the community level factors that bring near to death problem?
8. What are the organizational level factors that bring near to death problem?
9. What are policy level factors that bring near to death problem?
10. If you have additional idea, please express it
11. **Health extension workers, health care workers, and health office holders**
12. Please tell me the factors that you believe to bring this problem?
13. What are the individual level factors do you think are related with near to death experiences in this pregnancy?
14. Please explain the interpersonal factors that you believe for occurrence of near to death?
15. Please tell the community level factors that bring near to death problem?
16. What are the organizational level factors that bring near to death problem?
17. What are policy level factors that bring near to death problem?

If you have additional idea, please express it
